# Estimation of Total Usual Dietary Intakes of Pregnant Women in the United States

**DOI:** 10.1001/jamanetworkopen.2019.5967

**Published:** 2019-06-21

**Authors:** Regan L. Bailey, Susan G. Pac, Victor L. Fulgoni, Kathleen C. Reidy, Patrick M. Catalano

**Affiliations:** 1 Department of Nutrition Science, Purdue University, West Lafayette, Indiana; 2 Nestle Nutrition, Arlington, Virginia; 3 Nutrition Impact LLC, Battle Creek, Michigan; 4 Mother Infant Research Institute, Department of Obstetrics and Gynecology, Tufts University Medical Center, Boston, Massachusetts

## Abstract

**Importance:**

Nutrition during pregnancy is a critical dimension not only for women’s health but also for the lifelong health of the offspring. Very limited national data exist on the usual dietary intakes of pregnant women.

**Objective:**

To estimate total usual nutrient intakes (from foods and dietary supplements) and the population prevalence of meeting or exceeding the National Academies of Sciences, Engineering, and Medicine Dietary Reference Intake recommendations among pregnant US women.

**Design, Setting, and Participants:**

A cross-sectional analysis was performed of a nationally representative sample of 1003 pregnant US women aged 20 to 40 years from the 2001-2014 National Health and Nutrition Examination Survey.

**Exposure:**

Usual dietary intakes assessed by two 24-hour dietary recalls (including dietary supplements) adjusted for within-person variation using the National Cancer Institute method.

**Main Outcomes and Measures:**

The proportion of women at risk of inadequate dietary intake as determined by the Estimated Average Requirement, the proportion of women assumed to have adequate dietary intake as determined by the Adequate Intake, and the proportion of women at risk of excess dietary intake as determined by the Tolerable Upper Intake Level. Demographic differences between pregnant and nonpregnant women were compared with *t* tests.

**Results:**

As representative of the US population, this sample of 1003 pregnant women had a mean (SE) age of 28.0 (0.3) years, was predominantly non-Hispanic white (mean [SE], 54.5% [3.1%]), and was at above 185% of the income to poverty ratio (mean [SE], 56.8% [3.0%]). Most pregnant women used a dietary supplement (mean [SE], 69.8% [2.3%]). A total of 10% or more of pregnant women had a total usual intake that is less than the Estimated Average Requirement for magnesium (mean [SE], 47.5% [2.8%]), vitamin D (mean [SE], 46.4% [2.7%]), vitamin E (mean [SE], 43.3% [2.7%]), iron (mean [SE], 36.2% [2.8%]), vitamin A (mean [SE], 15.5% [2.1%]), folate (mean [SE], 16.4% [1.6%]), calcium (mean [SE], 12.9% [2.4%]), vitamin C (mean [SE], 11.5% [1.9%]), vitamin B_6_ (mean [SE], 11.5% [1.5%]), and zinc (mean [SE], 10.9% [1.9%]). Some pregnant women exceeded the Adequate Intake for potassium (mean [SE], 41.7% [2.9%]), choline (mean [SE], 7.9% [3.2%]), and vitamin K (mean [SE], 47.9% [4.3%]). Most women exceeded the Tolerable Upper Intake Level for sodium (mean [SE], 95.0% [2.2%]), and some women exceeded the Tolerable Upper Intake Level of folic acid (mean [SE], 33.4% [2.8%]), iron (mean [SE], 27.9% [2.8%]), calcium (mean [SE], 3.0% [0.8%]), and zinc (mean [SE], 7.1% [1.6%]). For iron, the prevalence of an at-risk intake from foods alone was lower among women who used supplements (mean [SE], 80.3% [4.3%]) than those who did not use supplements (mean [SE], 95.3% [7.3%]); however, supplement use increased the risk of excessive iron and folic acid intakes given the amounts that are being consumed from supplemental products.

**Conclusions and Relevance:**

This study suggests that a significant number of pregnant women are not meeting recommendations for vitamins D, C, A, B_6_, K, and E, as well as folate, choline, iron, calcium, potassium, magnesium, and zinc even with the use of dietary supplements. Almost all pregnant women in this study were at risk of excessive consumption of sodium, and many were at risk of excessive consumption of folic acid and iron. Improved dietary guidance to help pregnant women meet but not exceed dietary recommendations is warranted.

## Key Points

**Question:**

How do the usual dietary intakes of pregnant US women compare with the National Academies of Science, Engineering, and Medicine Dietary Reference Intakes for nutritional adequacy and excess?

**Findings:**

This cross-sectional analysis of 1003 pregnant US women found that many pregnant women did not consume enough key nutrients during pregnancy, specifically, vitamins A, C, D, E, K, and B_6_, as well as folate, choline, and minerals including iron, potassium, calcium, magnesium, and zinc. Almost all pregnant women consumed excessive sodium, and many were at risk of excessive consumption of folic acid and iron.

**Meaning:**

Improved dietary guidance appears to be warranted to help pregnant women to meet but not exceed dietary recommendations.

## Teaser

This cross-sectional study uses data from the 2001-2014 National Health and Nutrition Examination Survey (NHANES) to estimate total usual nutrient intakes from foods and dietary supplements of pregnant women.

## Introduction

Adequate food and nutrient intake during pregnancy is universally recognized as optimal for fetal development and maternal health.^1,2,3,4,5^ Increased caloric and nutrient intakes are recommended to meet the demands of the rapidly growing fetus and the increased physiological requirements of the mother, especially for folate, iron, iodine, and copper.^6,7^ Although nutrient intakes should preferably come from a variety of food sources, it is unlikely that pregnant women and those of childbearing age meet their needs for some nutrients through diet alone.^8,9^ As such, prenatal dietary supplements are generally recommended during pregnancy^10,11^ and were used by about 75% of pregnant women in a nationally representative US sample.^8^ However, prenatal dietary supplements provide variable nutrient content and the number of nutrients included in dietary supplement formulations is not standard.^12^ Concerns exist of excessive intakes of some nutrients during pregnancy, especially folic acid and vitamin A,^13,14^ while low intake of iron and iodine in reproductive-aged US women has also been of concern.^15^ Thus, ensuring that pregnant and reproductive-aged women have adequate, but not excessive, dietary intakes is crucial to understand how to best tailor public health messaging and policy during this critical life stage. The purpose of this study was to report the usual nutrient intakes from food and dietary supplements for pregnant women using data from the National Health and Nutrition Examination Survey (NHANES).

## Methods

### Participants and Data Collection

The NHANES is a nationally representative, cross-sectional survey of noninstitutionalized, civilian US residents using a complex, stratified, multistage probability cluster sampling design. The NHANES is conducted by the US Centers for Disease Control and Prevention National Center for Health Statistics, who obtained written informed consent for all participants or proxies. The NHANES survey protocol was approved by the National Center for Health Statistics Research Ethics Review Board. This analysis of secondary data was not subject to institutional review by any of the participating organizations. This analysis follows the Strengthening the Reporting of Observational Studies in Epidemiology (STROBE) guidelines.

Since 1999, the continuous NHANES survey protocol includes an in-person household interview, followed by a health examination in a mobile examination center, and a follow-up telephone interview. Pregnancy was determined from the demographics file (ie, RIDEXPRG = 1 “positive lab pregnancy test or self-reported pregnant at exam” from the mobile examination center). The NHANES data are released in 2-year cycles; because the sample size for pregnant women is very small, 7 survey cycles were combined providing estimates from 2001 to 2014. All nonlactating US adult women aged 20 to 40 years with reliable dietary data (as provided by the US Department of Agriculture [USDA] DR1SRSTZ and DR2SRSTZ variables in the NHANES data) were included and stratified by current pregnancy status: those who were pregnant and not lactating (n = 1003) and those who were not pregnant and not lactating (n = 5523).

During the in-person household interview, sociodemographic data (including age, sex, race/ethnicity, educational level, and family income to poverty ratio) were collected through a computer-assisted personal interview. Self-reported racial/ethnic groups, as defined in the NHANES and used in this analysis, were as follows: non-Hispanic white, non-Hispanic black, Hispanic and Mexican American, and other. Educational level was categorized as less than high school, high school diploma or equivalent or less, some college but no degree, and a college degree or higher. The income to poverty ratio, the ratio of annual family income to the poverty guideline, represented family income and was categorized as an income to poverty ratio of 130% or less, 131% to less than 185%, and 185% or more. The income to poverty ratio serves as an income eligibility criterion for some federal nutrition assistance programs; for example, an income to poverty ratio of less than 130% is the eligibility criterion for the Supplemental Nutrition Assistance Program.

Questions about smoking status and physical activity were also asked in the in-person household interview. Current smoking status was categorized as a dichotomous variable based on serum cotinine level (when available from data gathered at the mobile examination center) or self-report from the smoking questionnaire when the nicotine biomarker status was not available. Participants reported minutes per day and days per week spent in sedentary, moderate-intensity, and vigorous-intensity activities using the Physical Activity Questionnaire. Physical activity level was divided into 3 groups based on the number of days in which vigorous exercise was performed, using the Physical Activity Questionnaire responses (Physical Activity Questionnaire 560 for 2001-2008 and Physical Activity Questionnaire 706 for 2009-2014: sedentary, 0-3 days per week; moderate, 4-6 days per week; and vigorous, 7 days per week).

### Dietary Data

Dietary supplement information was measured via a questionnaire as part of a home inventory. Participants show the interviewer their supplement products when available (approximately 80% were available) and details are obtained on the participant’s use of vitamins, minerals, herbal supplements, and other supplements during the previous 30 days. Detailed information about type of supplement, consumption frequency, duration, and amount taken is also collected for each reported supplement and used to calculate mean daily intakes from all products. The NHANES Dietary Supplement Database provides information on the nutrient values of supplements reported by NHANES participants and contains label information from prescribed and over-the-counter products and nonprescription antacids containing calcium and/or magnesium. The current dietary supplement database provides product information in NHANES only from 1999 to 2014; thus, this report does not include 2015-2016 data as they are not available.

During the health examination in the mobile examination center, an in-person 24-hour dietary recall was collected as part of the What We Eat in America survey. A second 24-hour recall was collected via telephone 3 to 10 days after the first recall, with emphasis placed on obtaining both weekday and weekend reports. When weighted appropriately, dietary data represent both weekdays and weekend days. Both 24-hour recalls were collected using the USDA’s Automated Multiple-Pass Method.^16,17^ The USDA Food and Nutrient Database for Dietary Studies was used to convert foods and beverages as consumed into gram amounts and to determine their energy and nutrient values.^18^ The mean energy and macronutrient amounts (grams) as well as the percentage of energy contributed by each macronutrient were estimated. Given that energy intakes and dietary supplement use are higher during pregnancy, dietary intakes of pregnant and nonpregnant women were not statistically compared. Only usual nutrient intakes from foods and beverages and total usual intakes, inclusive of dietary supplements, of pregnant women are presented in this study. The USDA and others have published extensively on NHANES data regarding the dietary intakes of nonpregnant US women in these age groups, with data available from 1988 to the present.^19,20,21,22,23,24^

The bioavailability of folate in food is assumed to be lower than that of folic acid present in fortified foods and dietary supplements. For this reason, the dietary folate equivalent conversion was developed to reflect the differential bioavailability.^25^ The dietary folate equivalent is used to estimate nutrient adequacy, but only the folic acid form is used to assess the potential risk of excess. Similarly, carotenoids have vitamin A activity and can be used to meet the recommendations for Adequate Intake; however, preformed retinol is the only form of vitamin A considered for potential excess. Iodine is not available in the USDA Food and Nutrient Database for Dietary Studies and is not included in this analysis; information on urinary iodine concentrations and iodine from dietary supplements alone has been previously published.^26^ Data on dietary choline intake are not available in all survey years; estimates are presented only from 2005 to 2014. Excessive niacin intake could not be estimated, as the form of niacin (ie, nicotinic acid) used to assess the Upper Tolerable Level (UL) is not available in the federal databases. Data on vitamins A and E from dietary supplements were estimated for 2007 and beyond using previous databases of products, as this information is not currently available in the NHANES.

### Statistical Analysis

Before a diet can be characterized as at risk for inadequacy or excess relative to Dietary Reference Intakes, usual or long-term estimates are needed that are adjusted for random measurement error (ie, day-to-day variation) from daily self-reported diet assessments.^27,28,29^ The 24-hour dietary recall provides a relatively unbiased measure of intake on a given day but, because of high within-person variation, cannot provide reliable estimates of usual nutrient intakes.^30,31,32^ For this reason, several procedures have been developed to estimate the distribution of usual intakes when only a small number of 24-hour dietary recalls are available per individual.^33,34,35,36,37^ These methods use statistical modeling to approximate the distribution that would be obtained by averaging many 24-hour dietary recalls per person. All these methods have a similar underlying framework that a single day of intake is not representative of usual or habitual intakes and thus seek to remove random error to the extent possible, and require at least 2 repeated measurements for a representative subsample of the population group of interest to allow computation of both variance components.^38^ Complete details of how usual total nutrient intakes are estimated is published elsewhere.^38,39^

For this this analysis, macros developed to implement the National Cancer Institute method,^36,40^ using the single component models as most nutrients are consumed by most individuals on each day, were used to produce the mean and SE for a given usual intake, as well as the population prevalence that met the Estimated Average Requirement (EAR) and exceeding the Adequate Intake (AI) or Tolerable Upper Intake Level (UL), using the cut-point approach. The cut-point method proposed by the National Academy of Science, Engineering, and Medicine (formerly the Institute of Medicine) was used for all nutrients, including iron. The cut-point method provides an estimate of the proportion of individuals in the group who are at risk for inadequate intakes and excessive intakes.^38^ For nutrients without an EAR (eg, vitamin K, sodium, potassium, and choline), the percentage of individuals with usual intake above AI was also determined using the cut-point method. Covariates for usual intake determination were day of the week of the 24-hour recall (coded as weekend [Friday-Sunday] or weekday [Monday-Thursday]), sequence of dietary recall (first or second), and whether dietary supplements were consumed (yes or no). Balanced repeated replication was performed to obtain SEs; balanced repeated replication weights were constructed with Fay adjustment factor M = 0.3 (perturbation factor, 0.7) and further adjusted to match the initial sample weight totals within specific age, sex, and race/ethnic groupings for the full data set. Statistical analyses were performed using SAS, version 9.3 (SAS Institute Inc) and SUDAAN, version 11.1 (RTI) software.

Given pregnant women’s higher energy intakes and use of dietary supplements, their dietary intakes were not statistically compared with those of nonpregnant women for micronutrient intakes. However, demographic and lifestyle data presented in Table 1 were compared using *t* tests. Sampling weights and the sampling units and strata information, as provided by the NHANES, were included in all analyses. Point estimates with a relative SE of greater than 30% are not displayed, as outlined by the National Center for Health Statistics analytical guidelines.^41^ Two-tailed *P* < .05 was considered statistically significant.

**Table 1.** Sample Characteristics for US Women Aged 20 to 40 Years by Pregnancy Status in the National Health and Nutrition Examination Survey, 2001-2014

Characteristic	Nonpregnant and Nonlactating Women, Mean (SE) (n = 5523)	Pregnant and Nonlactating Women, Mean (SE) (n = 1003)
Age, y	30.0 (0.2)	28.0 (0.3)^a^
Trimester of pregnancy, %		
First	NA	26.5 (2.5)
Second	NA	36.0 (3.1)
Third	NA	37.5 (3.0)
Race/ethnicity, %^b^		
Non-Hispanic white	62.0 (1.6)	54.5 (3.1)^a^
Hispanic or Mexican	17.2 (1.1)	19.7 (2.3)
Non-Hispanic black	13.8 (0.9)	17.5 (2.2)
Educational level, %		
High school or GED or less	36.9 (1.2)	39.2 (2.6)
Some college but no degree	37.3 (0.9)	31.5 (2.3)^a^
Undergraduate degree or higher	25.8 (1.0)	29.2 (2.6)
Income to poverty ratio, %		
≤130	31.0 (1.1)	29.8 (2.3)
131 to <185	11.8 (0.5)	13.4 (1.7)
≥185	57.2 (1.1)	56.8 (3.0)
Physical activity, %^c^		
Sedentary	21.9 (0.8)	33.3 (2.8)^a^
Moderate	35.0 (1.0)	48.9 (2.8)^a^
Vigorous	43.1 (1.3)	17.8 (2.1)^a^
Dietary supplement use, %	47.1 (1.1)	69.8 (2.3)^a^
Alcohol use, %	21.7 (0.9)	3.4 (0.7)^a^
Smoking currently, %	20.3 (0.9)	5.9 (1.1)^a^

Abbreviations: GED, general equivalency diploma; NA, not applicable.

^a^ Means or proportions are significantly different (*P* < .05).

^b^ Race/ethnicity does not sum to 100% because the “other” category is not presented per National Center for Health Statistics analytical guidelines.

^c^ Physical activity level was divided into 3 groups based on number of days in which there was hard exercise using the Physical Activity Questionnaire responses.

## Results

As representative of the US population, this sample of 1003 pregnant women had a mean (SE) age of 28.0 (0.3) years, was predominantly non-Hispanic white (man [SE], 54.5% [3.1%]), and was at or above 185% of the income to poverty ratio (mean [SE], 56.8% [3.0%]) (Table 1). Fewer non-Hispanic white women and women with a high school diploma or equivalent and less than a college education were currently pregnant during these survey years than those who were not; other racial/ethnic, educational attainment, and income to poverty ratio differences were not statistically significant between the pregnancy status groups. Pregnant women tended to have lower levels of vigorous physical activity (and more sedentary physical activity) and reported less current smoking and alcohol consumption, but they were more likely to use a dietary supplement (mean [SE], 69.8% [2.3%]). Energy and macronutrient distributions of pregnant women are presented in Table 2; pregnant women had higher mean energy intake than did nonpregnant women (2232 vs 1928 kcal).

**Table 2.** Data on Total Usual Intakes of Energy and Macronutrients for US Women Aged 20 to 40 Years by Pregnancy Status in the National Health and Nutrition Examination Survey, 2001-2014

Dietary Component	Nonpregnant and Nonlactating Women, Mean (SE) (n = 5523)	Pregnant and Nonlactating Women, Mean (SE) (n = 1003)
Energy, kcal/d	1928 (11)	2232 (42)
Carbohydrate, g/d	242 (1.7)	294 (7.0)
% of Total energy	50.1	52.1
Added sugars, tsp/d	18.9 (0.3)	21.2 (0.9)
% of Total energy	15.4	14.3
Total fat, g/d	72.5 (0.7)	83.6 (2.1)
% of Total energy	32.7	33.0
Saturated fat, g/d	23.9 (0.2)	28.3 (0.8)
% of Total energy	10.7	11.1
Protein, g/d	71.4 (0.6)	81.9 (1.8)
% of Total energy	15.1	14.9
Alcohol, g/d	7.2 (0.4)	0.6 (0.2)
Dietary fiber, g/d	14.2 (0.2)	17.3 (0.5)

The risk of dietary inadequacy was lower for many nutrients based on total intake (from both food and dietary supplements) when compared with intakes from food alone (Table 3). For example, the mean (SE) population prevalence of those at risk of dietary inadequacy for vitamin A was 27.7% (4.2%) from foods alone, which was reduced to 15.5% (2.1%) when dietary supplements were included. This risk reduction with the use of dietary supplements was the case for most nutrients examined. However, for some nutrients that are not common in dietary supplements (ie, sodium and potassium), are found in low amounts in dietary supplements used by pregnant women (ie, choline), or are consumed in adequate amounts from foods alone (ie, phosphorus and selenium), few differences were observed between the population prevalence of women at risk of dietary inadequacy from foods and beverages alone and total intakes.

**Table 3.** Data on Usual Nutrient Intake Distributions From Foods and Beverages Alone and Total Intakes From Foods and Supplements and Prevalence Estimates Relative to the Dietary Reference Intake Recommendations of Pregnant Women Aged 20 to 40 Years in the National Health and Nutrition Examination Survey, 2001-2014

Nutrient	EAR [AI]^a^	UL	Foods Alone				Foods and Supplements			
			Mean (SE)	% <EAR	% >AI	% >UL	Mean (SE)	% <EAR	% >AI	% >UL
Vitamin A, μg/d^b^^,^^c^	770	3000	696 (27)	27.7 (4.2)	NA	0	1283 (54)	15.5 (2.1)	NA	ES^d^
Thiamin, mg/d	1.2	NA	1.8 (0.4)	11.5 (2.9)	NA	NA	3.6 (0.3)	5.7 (1.4)	NA	NA
Riboflavin, mg/d	1.2	NA	2.3 (0.05)	5.0 (1.5)	NA	NA	4.1 (0.3)	3.0 (0.8)	NA	NA
Niacin, mg/d^e^	14	35	24.4 (0.6)	2.8 (1.5)	NA	NA	35.2 (1.2)	1.3 (0.7)	NA	NA
Vitamin B_6_, mg/d	1.6	100	2.1 (0.1)	25.4 (3.2)	NA	0	7.8 (0.7)	11.5 (1.5)	NA	ES^d^
Folate, μg DFE/d^f^	520	1000	630 (25)	35.8 (3.4)	NA	0	1451(51)	16.4 (1.6)	NA	33.4 (2.8)
Choline^g^	[450]	3500	321 (10)	NA	7.7 (3.1)	0	322 (10.6)	NA	7.9 (3.2)	0
Vitamin B_12_, μg/d	2.2	NA	5.6 (0.2)	2.4 (1.2)	NA	NA	19.2 (6.4)	1.4 (0.6)	NA	NA
Vitamin C, mg/d	70	2000	122 (6)	24.7 (3.4)	NA	0	199 (9.4)	11.5 (1.9)	NA	ES^d^
Vitamin D, μg/d	10	100	5.5 (0.2)	92.1 (1.8)	NA	0	11.3 (0.4)	46.4 (2.7)	NA	ES^d^
Vitamin E, mg/d^b^^,^^h^	12	1000	7.8 (0.8)	91.8 (2.5)	NA	NA	21.0 (1.3)	43.3 (2.7)	NA	NA
Vitamin K, μg/d	[90]	NA	98.2 (5)	NA	46.9 (4.4)	NA	99.5 (5.1)	NA	47.9 (4.3)	NA
Calcium, mg/d	800	2500	1093 (28)	21.2 (3.7)	NA	ES^d^	1311 (35)	12.9 (2.4)	NA	3.0 (0.8)
Iron, mg/d	22	45	17.2 (0.5)	83.8 (3.9)	NA	ES^d^	38.3 (2.0)	36.2 (2.8)	NA	27.9 (2.8)
Magnesium, mg/d^i^	290	350	294 (6.4)	53.3 (3.0)	NA	NA	314 (7.2)	47.5 (2.8)	NA	ES^d^
Selenium, μg/d	49	400	110 (2.7)	ES^d^	NA	0	114 (2.9)	ES^d^	NA	0
Phosphorus, mg/d	580	3500	1412 (29)	ES^d^	NA	0	1422 (29)	ES^d^	NA	0
Copper, mg/d	0.8	10	1.4 (0.03)	5.4 (1.9)	NA	0	1.8 (0.1)	4.5 (1.4)	NA	0
Zinc, mg/d	9.5	40	12.4 (0.4)	21.5 (4.0)	NA	0	22.7 (0.8)	10.9 (1.9)	NA	7.1 (1.6)
Sodium, mg/d	[1500]	2300	3637 (82)	NA	99.9 (0.2)	93.6 (2.4)	3639 (84)	NA	99.9 (0.2)	95.0 (2.2)
Potassium, mg/d	[2900]	NA	2778 (58)	NA	41 (2.8)	NA	2786 (58)	NA	42 (2.9)	NA

Abbreviations: AI, Adequate Intake; DFE, dietary folate equivalent; EAR, Estimated Average Requirement; ES, estimate suppressed; NA, not applicable; UL, Tolerable Upper Intake Level.

^a^ The AI is presented in brackets because it reflects that population prevalence is greater than the AI.

^b^ Dietary supplement data were not available for survey years 2007 to 2014 and were estimated using previous databases of products.

^c^ The EAR is set based on retinol activity equivalents and the UL is set based on preformed retinol only.

^d^ Estimate suppressed because of relative SE greater than 30% given that less than 0.5% of the population is represented.

^e^ The UL for niacin is based on nicotinamide and data on intake on this form are not available.

^f^ The EAR is set based on DFE; the UL is set based on folic acid form only in fortified foods and dietary supplements.

^g^ The sample size available for choline is 533.

^h^ The EAR is only for alpha tocopherol and 4 stereoisomers that occur in fortified foods and supplements; the ULs apply to all forms of supplemental alpha tocopherol, including the 8 stereoisomers present in synthetic vitamin E.

^i^ The EAR is 290 mg/d in pregnancy for ages 19 to 30 years and 300 mg/d for ages 31 to 50 years. The UL for magnesium is only from supplemental sources and pharmacologic products.

Less than 10% of pregnant women were at risk for inadequate total intake of thiamin (mean [SE], 5.7% [1.4%]), riboflavin (mean [SE], 3.0% [0.8%]), niacin (mean [SE], 1.3% [0.7%]), vitamin B_12_ (mean [SE], 1.4% [0.6%]), copper (mean [SE], 4.5% [1.4%]), phosphorus (mean, <0.5%; estimate suppressed because of high relative SE) and selenium (mean, <0.5%; estimate suppressed because of high relative SE) (Table 3). Even after accounting for dietary supplement use, at least 10% of pregnant women had a total usual intake less than the EAR for magnesium (mean [SE], 47.5% [2.8%]), vitamin D (mean [SE], 46.4% [2.7%]), vitamin E (mean [SE], 43.3% [2.7%]), iron (mean [SE], 36.2% [2.8%]), vitamin A (mean [SE], 15.5% [2.1%]), folate (mean [SE], 16.4% [1.6%]), calcium (mean [SE], 12.9% [2.4%]), vitamin C (mean [SE], 11.5% [1.9%]), vitamin B_6_ (mean [SE], 11.5% [1.5%]), and zinc (mean [SE], 10.9% [1.9%]). A mean (SE) of 41.7% (2.9%) of pregnant women had a usual intake higher than the AI for potassium and 7.9% (3.2%) had a usual intake higher than the AI for choline, whereas 47.9% (4.3%) had a usual intake higher than the AI for vitamin K. Most pregnant women (mean [SE], 95.0% [2.2%]) exceeded the UL for sodium, while some exceeded the UL for folic acid (mean [SE], 33.4% [2.8%]), iron (mean [SE], 27.9% [2.8%]), calcium (mean [SE], 3.0% [0.8%]), and zinc (mean [SE], 7.1% [1.6%]). Aside from sodium, few pregnant women exceeded the UL from food sources alone (<0.5% for calcium and iron; estimate suppressed because of high relative SE). Similarly, less than 0.5% of pregnant women exceeded the UL for retinol; vitamins B_6_, C, and D; and magnesium.

Given the large contribution that dietary supplements make toward iron and folic acid in pregnant women and the fact that, as a group, many pregnant women were exceeding the UL of these nutrients, we further stratified the analysis by users and nonusers of dietary supplements (Figure). When users of dietary supplements were examined separately, approximately 5% had a usual intake less than the EAR but almost half (47.7%) also exceeded the UL. For iron, the prevalence of an at-risk intake from foods alone was lower among women who used supplements (mean [SE], 80.3% [4.3%]) than those who did not use supplements (mean [SE], 95.3% [7.3%]), which was considerably reduced when supplements were included in estimates (mean [SE], 13.9% [2.3%]). Similar to folate, no women exceeded the UL for iron from diet alone, but among women who used dietary supplements, a mean (SE) of 40.2% (3.5%) did so.

**Figure.**
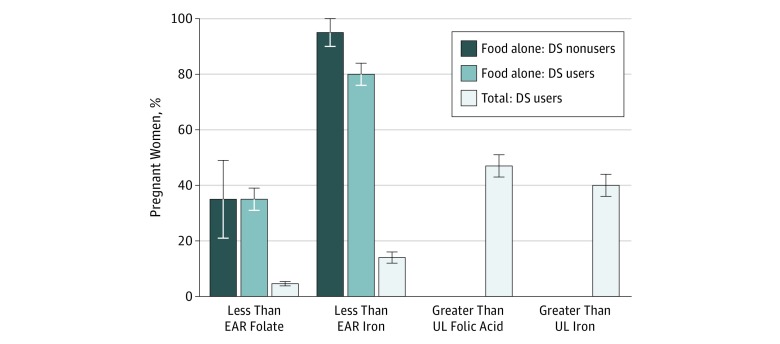
Prevalence of Usual Nutrient Intake Distributions From Food Alone and Total Intake Below the Estimated Average Requirement (EAR) and Above the Tolerable Upper Intake Level (UL) for Folate and Iron for Nonlactating Pregnant Women Aged 20 to 40 Years Data are from 1003 women participating in the National Health and Nutrition Examination Survey, 2001-2014. The EAR is set based on the dietary folate equivalent that includes natural and synthetic forms; the UL is set based on synthetic folic acid form only in fortified foods and dietary supplements (DS). The prevalence of exceeding the UL from food sources alone is 0 for folate and iron among users and nonusers of dietary supplements. The error bars indicate the SEs.

## Discussion

Nutrition during the first 1000 days of life (including in utero exposures) has emerged as a critical dimension of lifelong health and well-being.^2,42,43,44,45^ Thus, for the first time the 2020-2025 Dietary Guidelines for Americans will include nutrition recommendations for women during pregnancy and lactation, as well as for children from birth to age 24 months.^46^ Given the very specific nutritional needs during pregnancy to support the growth and development of the fetus, understanding contemporary dietary intakes during pregnancy is critical to inform such policy and best tailor dietary advice for women and their clinicians. Despite the importance of perinatal nutrition, very limited domestic data are available on the dietary intakes of pregnant women.^8^ Historically, the sample sizes from the NHANES have not been sufficient to make statistically reliable estimates. During a period from 2001 to 2006, pregnant women were oversampled in NHANES and, when combined with more recent NHANES survey waves, adequate sample size is available across 14 years of NHANES cycles to provide reliable estimates for the dietary intakes of pregnant US women for most nutrients. A 2013 systematic review and meta-analysis by Blumfield et al^5^ of the dietary intakes of pregnant women in developed countries identified suboptimal iron, folate, and vitamin D among women from all countries, with calcium also being a concern in women from Japan. The report by Blumfield et al,^5^ similar to this report, lacked sufficient data to make estimates of iodine intakes. Iodine is a particular nutrient of concern during pregnancy because the iodine requirement increases by more than 50% during pregnancy secondary to fetal needs, alterations in maternal iodine metabolism, and enhanced renal clearance of iodine.^26^ Iodine contributions from foods are not available in the NHANES^47^ because estimating the iodine content of foods is difficult owing to its variability in the soil. A previous analysis of NHANES 1999-2006 indicated that only about 23% of prenatal supplements used by pregnant US women contain any iodine.^26^ Public health monitoring efforts have used urinary iodine concentration to estimate the risk of inadequate dietary intake and suggest that iodine intakes of reproductive-aged US women remains low.^15,26^ The mean urinary iodine content among women in the NHANES was 148 μg/L (to convert to nanomoles per liter, multiply by 7.880), which is lower than the World Health Organization cutoff for insufficient iodine (<150 μg/L).^26^

Similarly, little is known about the choline and ω-3 fatty acid intakes during pregnancy. Recent work has also suggested that ω-3 fatty acids and choline are critical nutrients during the perinatal period. In a recent Cochrane review, ω-3 fatty acids (from fish and dietary supplements) were associated with lower risk of preterm birth and low birth weight but with a slightly increased risk of babies large for gestational age.^48^ Data on the choline intakes of the US population are available only for more recent NHANES survey cycles. Only 8% of pregnant women have an intake that meets the AI for choline intake, which has been recognized for its role in fetal neural development^49^; choline requirements may be higher in the third trimester.^50^ At this time, choline content in prenatal supplements is inconsistent in the United States, with some supplements containing either no choline or only small amounts of choline (40-55 mg).^51^ More guidance to pregnant women about increasing their intake of choline-rich foods may be warranted in addition to more consistency in the amount of choline provided in prenatal supplements.^52^

This analysis of usual nutrient intakes for pregnant women conducted using data from NHANES is the first of its kind in recent years, to our knowledge. Differences in sample characteristics between pregnant and nonpregnant women include behavior changes associated with public health recommendations for pregnant women such as smoking cessation and use of dietary supplements. Although prenatal dietary supplements are routinely recommended or prescribed during prenatal care, 30% of women in this analysis did not report using any dietary supplements during pregnancy. Among those who took dietary supplements, the supplements were most likely to be prenatal dietary supplements presented by a health care practitioner.^8^ During pregnancy, nutrient requirements increase for many nutrients but most notably for iron, folate or folic acid, iodine, and zinc, and dietary supplement use is often encouraged.^53^ The intake of iron and the intake of folic acid in this analysis share a unique pattern. For both of these nutrients, higher amounts are recommended during pregnancy. Many prenatal supplements in the United States contain 100% of the daily value for pregnant women for both folic acid and iron. Iron deficiency during pregnancy is associated with low birth weight, preterm delivery, and increased perinatal infant and maternal mortality. Anemia has been identified in approximately 9% of pregnant US women, with rates highest in non-Hispanic black pregnant women.^54^ Adequate folic acid exposure early in pregnancy is associated with a reduced risk of birth defects.^4^ Without the use of dietary supplements, most women fail to achieve the recommendations for iron intake (80%-95%) and about one-third fail to meet recommendations for folate intake (35%-36%); however, use of a dietary supplement substantially increases the intake for both these nutrients beyond the UL. As no women exceed the UL from foods alone, these data could be used to help health care practitioners guide the choice of dietary supplement based on the amount of nutrients that are necessary.

### Strengths and Limitations

The strengths of this study include new population-based data on the total usual nutrient intakes from foods and dietary supplements among a nationally representative sample of pregnant women. The major limitation of this cross-sectional survey is that diet was measured by self-report, which is subject to both random and systematic measurement error. Usual intake means that single-day estimates of intake are adjusted for random measurement error; this adjustment is particularly important when looking at the tails of the distributions or the prevalence of individuals at risk for inadequacy or excess.^38^ We combined users and nonusers of dietary supplements to provide national estimates during pregnancy; however, data presented this way tend to underestimate nutritional exposures for supplement users and overestimate nutritional exposures for nonusers.^20,55,56^ Specifically, 30% of women do not use a dietary supplement during pregnancy, so the estimates of nutrient inadequacy are likely higher among these women. Most of the dietary supplements used in this population group do not contain fiber, sodium, macronutrients, or potassium. We have limited this report to the most recently available data on nutrients from foods and supplements; currently, the NHANES 2015-2016 has only nutrient estimates from foods.

Most available Dietary Reference Intakes were set between 1997 and 2005, and only 2 nutrients have been updated since then: calcium and vitamin D in 2011.^57^ Little experimental research is available on nutrient requirements during pregnancy. An AI is assumed to exceed the Recommended Daily Allowance for a nutrient, if one could be established. Thus, in applying the AI, the proportion of a group that exceeds the AI should reflect those who have adequate intakes, but there is no scientific basis to state that the proportion of intakes lower than the AI is an estimate of the prevalence of inadequacy. Thus, future work is needed to better define vitamin K, choline, and potassium requirements during pregnancy.

## Conclusions

This study indicates that dietary supplements appear to help pregnant women meet recommendations to increase intake of some key nutrients, and that most pregnant women take a dietary supplement. Although inconsistent definitions of multivitamin-mineral dietary supplements exist,^58,59^ most dietary supplements consumed during pregnancy include folic acid and iron.^8,12^ The number of nutrients and the amounts provided by prenatal supplements vary markedly depending on whether the product was obtained via a prescription or over the counter.^12^

The study suggests that a significant number of pregnant women are not meeting recommendations for some essential nutrients—vitamins D, C, A, K, and E, as well as iron, folic acid, calcium, potassium, magnesium, and choline—even with the use of dietary supplements. The use of dietary supplements reduces inadequate intakes but also increases the percentage of pregnant women with an intake above the UL. Because pregnant women do not exceed the UL with intakes from foods and beverages, dietary supplements play a role in some pregnant women exceeding the UL for these nutrients. It appears that supplements may be necessary for most pregnant women to meet nutrient recommendations; however, our findings suggest that responsible formulations of prenatal products could help women achieve recommended intakes without the potential for excess. In addition, similar to the general US population, sodium intakes during pregnancy were very high, with almost all women exceeding the UL. Improved dietary guidance to help pregnant women meet dietary recommendations for essential nutrients appears to be warranted.
